# Supplementary material for: Neural Organoids Protect Engineered Heart Tissues From Glucolipotoxicity by Transferring Versican in a Co‐Culture System
Source: Cell Prolif. 2025 Jun 3;59(1):e70070. doi: 10.1111/cpr.70070 (PMC12774625; doi:10.1111/cpr.70070)
Supplement: Supplementary file 1 — Data S1. Supporting Information. [file CPR-59-e70070-s004.pdf]

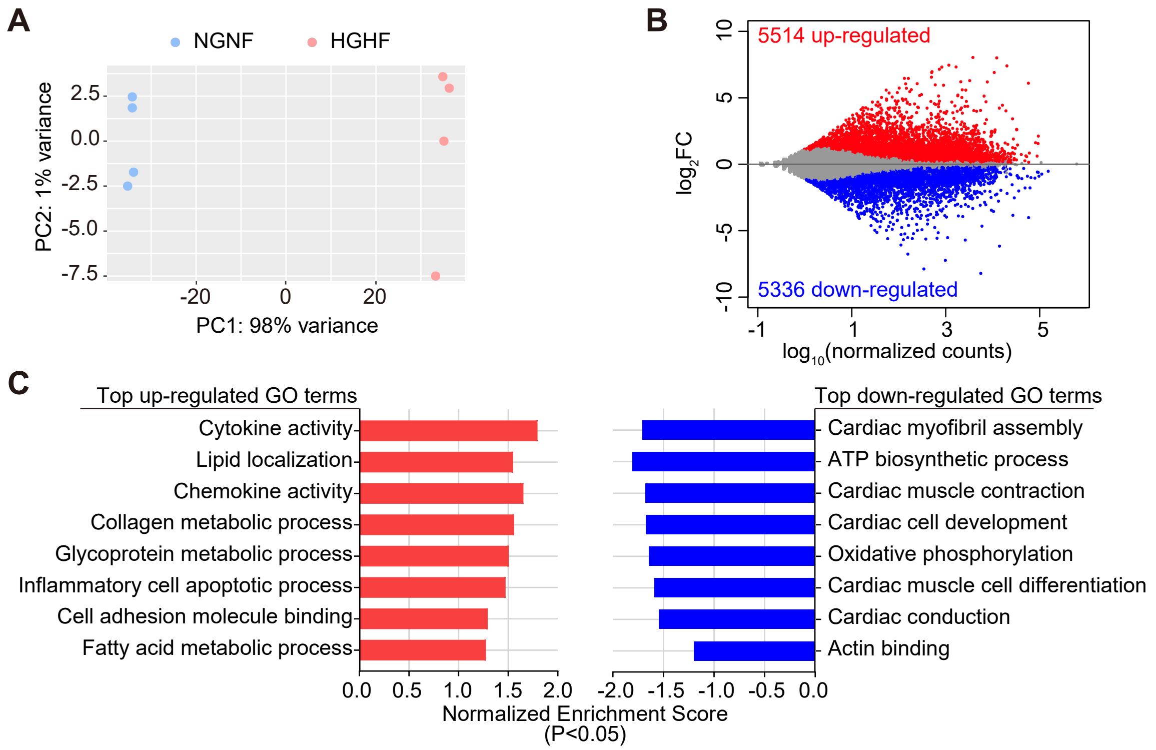

Figure S1. RNA-seq analysis of glucolipotoxicity in rat EHTs. A) PCA plot showing segregation of RNA-seq data. Rat EHTs were treated with NGNF or HGHF medium for 2 days. B) MA plot showing differentially expressed genes (Padj<0.05). C) GSEA analysis of differentially regulated GO terms in rat EHTs treated with NGNF or HGHF medium for 2 days.

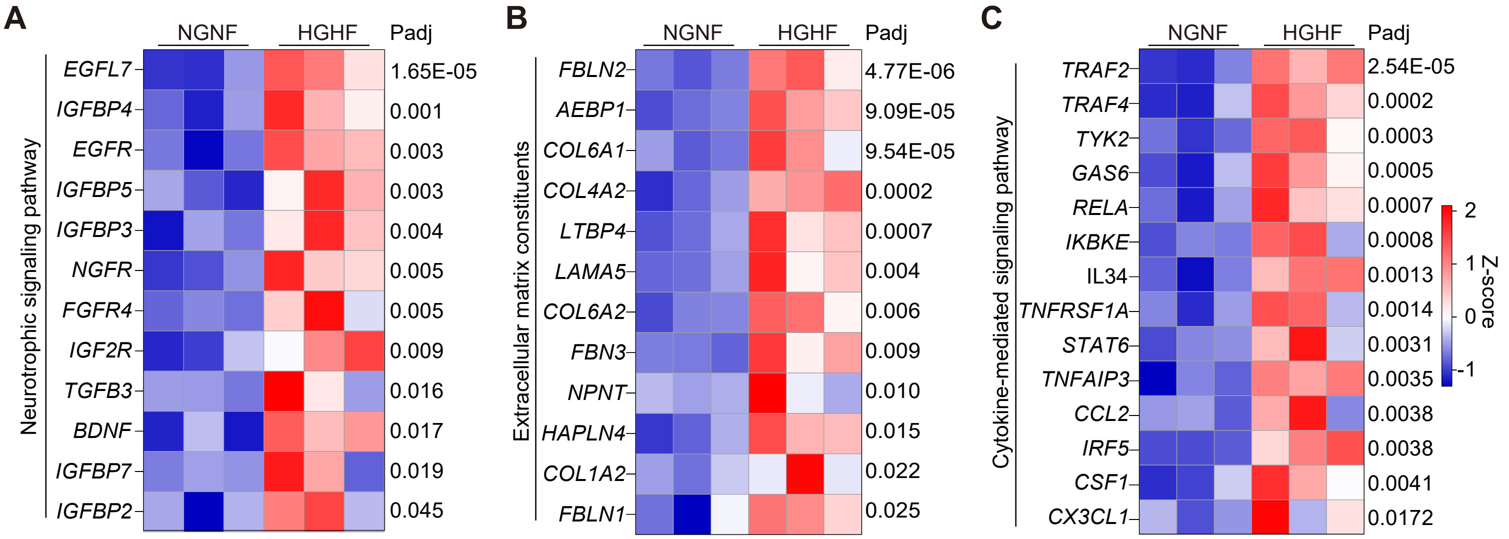

Figure S2. Heatmap of Smart-seq analysis of NOs treated with NGNF or HGHF medium for 2 days. A) Neurotrophic signaling pathway. B) Extracellular matrix constituents. C) Cytokine signaling pathways.

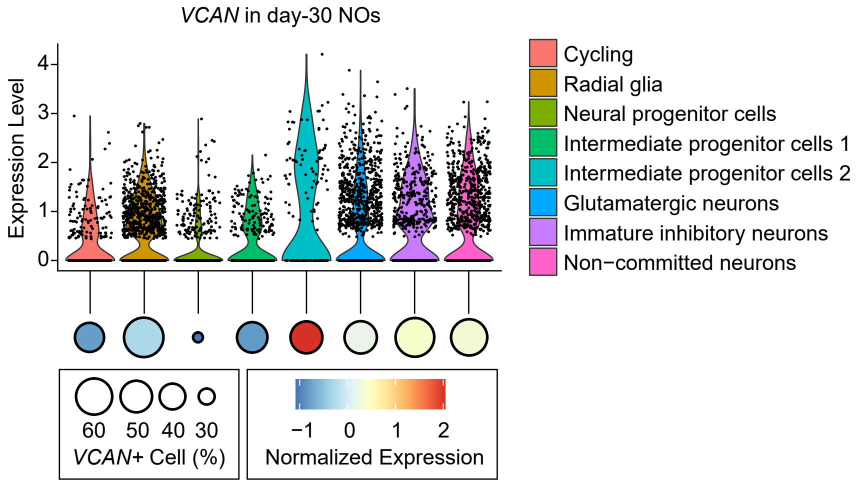

Figure S3. Single-cell RNA-seq analysis of *VCAN* in day-30 NOs. The violin plot and dot plot shows the expression levels of *VCAN* gene in different cell clusters. The bubble plot shows the proportion of *VCAN*-positive cells and the the expression levels of *VCAN* gene.

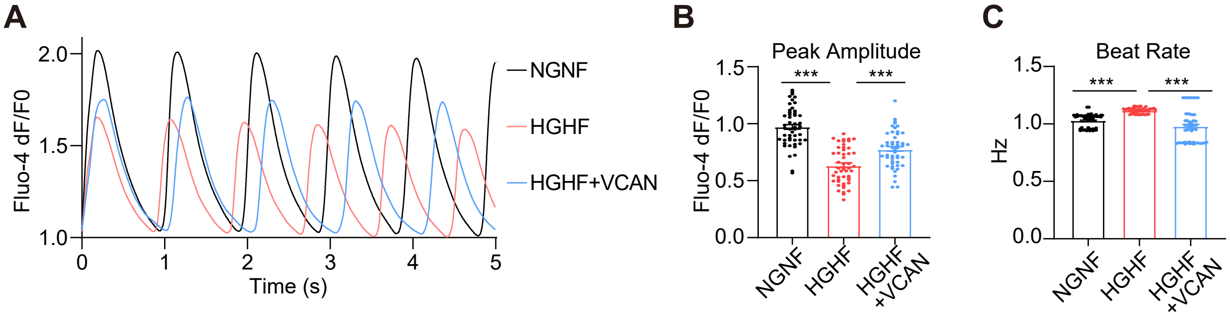

Figure S4. The effect of versican on cardiomyocyte calcium transient. A) Average Ca^{2+} transeint signals of neonatal rat ventricular cardiomyocytes under autonomous beating. B) Analysis of Ca^{2+} transeint peak amplitudes of NRVMs. C) Cardiomyocyte beat rate analysis. Data are plotted as mean ± SEM and analyzed by Mann-Whitney test (B, C), n = 50 cells per group, ***$P<0.001$.

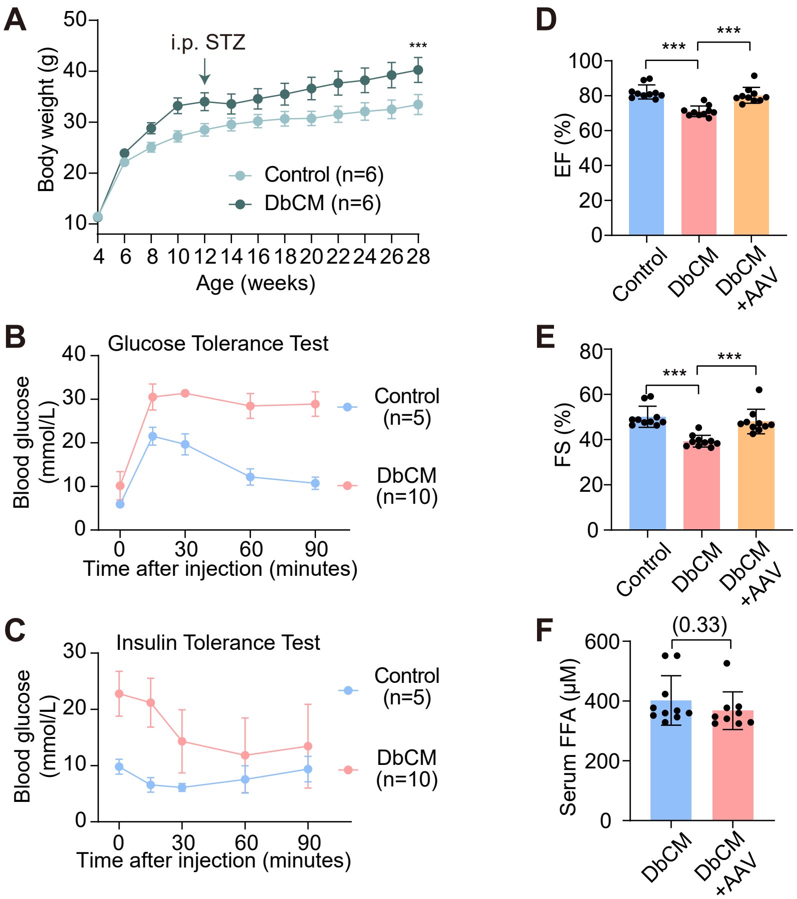

Figure S5. Basic characterization of DbCM mice. A) Body weight changes of mice in the control and DbCM group. B-C) Changes in blood glucose metabolism was detected by intraperitoneal glucose tolerance test (B) and intraperitoneal insulin tolerance test (C). D-E) Echocardiogram analysis of ejection fraction (EF) (D) and fractional shortening (FS) (E). F) Quantifcation of serum free fatty acids (FFA) concentration in DbCM and DbCM+AAV groups. Data are plotted as mean ± SEM and analyzed by two-way ANOVA with Tukey's post hoc test (A), one-way ANOVA with Tukey's post hoc test (D, E) or unpaired t test (F). *P < 0.05; **P < 0.01; ***P < 0.001.

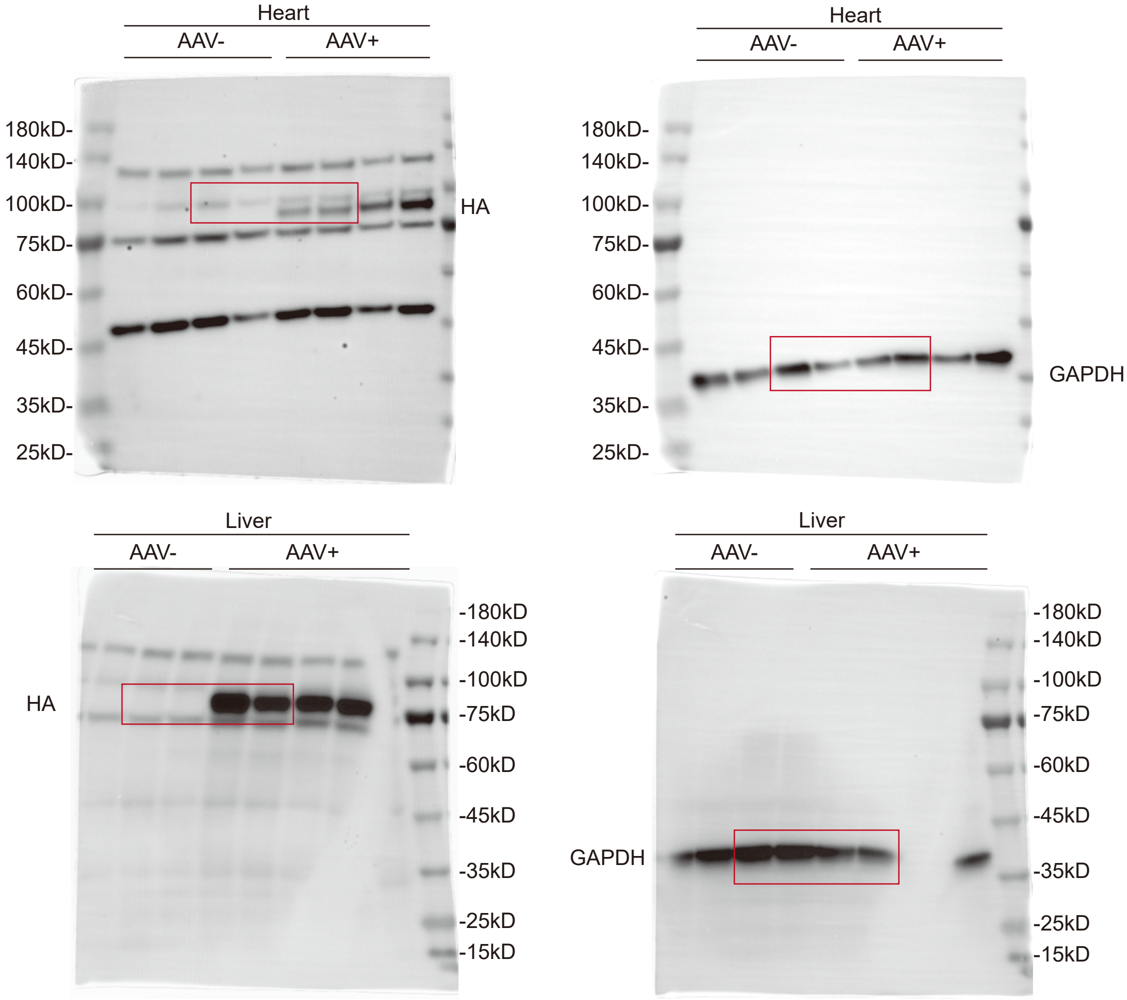

Figure S6. Uncropped Western blot images for Figure 8E.

Supplementary Table S1. The key reagents for engineered heart tissues (EHT) and neural organoids (NO) construction.

Reagent	Use	Supplier	Cat. No.
Dispase II	Digestion of hESCs (0.6 mg/mL)	Solarbio	D6430
SB431542	Neural induction (2 μM)	Topscience	T1726
Dorsomorphin homolog 1	Neural induction (2 μM)	Topscience	T1942
N2 Supplement (100×)	Neural induction (1×)	Gibco	17502001
Non-Essential Amino Acids (100×)	Medium supplement (1×)	Procell	PB180424
Fetal bovine serum	Medium supplement (5%/10%)	VISTECH	SE100-011
B27 Supplement Minus Vitamin A (50×)	Medium supplement (1×)	Gibco	12587001
6-aminoacetic acid	Plasmin inhibitors (2 mg/ml)	Sigma	A2504
L-ascorbic acid	Antioxidant (0.4 mg/mL)	Sigma	A8960
Thrombin	Activation of fibronectin (50 U/mL)	Sigma	T6884
Matrigel	Composition of hydrogel (10%)	Corning	354277
Fibronectin	Composition of hydrogel (2 mg/mL)	Sigma	F3879
Pluronic F-127	Non-ionic copolymer surfactant (2‰)	Sigma	P2443
Vitamin B12	Antioxidant (2 μg/mL)	Sigma	v6629
DMEM (low glucose)	Basic medium	Thermo	31600034
DMEM/F12 medium	Basic medium	Procell	PM150313
DMEM (high glucose)	Basic medium	HUANKE	HK2109.07
Brdu	Medium supplement (100 μM)	Bestbio	BB-4261
Y27	Medium supplement (10 μM)	Selleck	S1049
Collagenase Type 2	Primary cell isolation (0.056%)	Worthington	LS004176
Trypsin	Primary cell isolation (0.1%)	Solarbio	T8150
Hank's Balanced Salt Solution	Rinsing of tissue blocks	Procell	PB180321

Supplementary Table S2. Antibodies information for immunofluorescence.

Antibody	Use	Supplier	Cat. No.
[rabbit]Anti-FOXG1	IF (1:200)	Abcam	ab196868
[mouse]Anti-MAP2	IF (1:200)	Sigma	M1406
[rabbit]Anti-VCAN	IF (1:200)	Thermofisher	PA1-1748A
[mouse]Anti-TNNT2	IF (1:200)	Abcam	ab8295
[rabbit]Anti-TNNT2	IF (1:200)	Abcam	ab209813
[rabbit]Anti-Ki67	IF (1:200)	Abcam	ab267372
**Secondary Antibody**	**Use**	**Supplier**	**Cat. No.**
Anti-Rabbit IgG, Alexa Fluor-488	IF (1:500)	Abcam	ab150081
Anti-Mouse IgG, Alexa Fluor-555	IF (1:500)	Abcam	ab150114
**Reagent**	**Use**	**Supplier**	**Cat. No.**
DAPI	IF (1:1000)	Abcam	ab285390

Supplementary Table S3. The primer sequences for RT-qPCR.

Name	Nucl acid sequence (5'-->3')
Hsa_GAPDH-F	GATGACATCAAGAAGGTGGTGA
Hsa_GAPDH-R	GTCTACATGGCAACTGTGAGGA
Hsa_FOXG1-F	AGGAGGGCGAGAAGAAGAAC
Hsa_FOXG1-R	TGAACTCGTAGATGCCGTTG
Hsa_MAP2-F	TCCAAATGTGGCTCTCTGAA
Hsa_MAP2-R	TAGCTTGGGCCTTTTCTTTG
Hsa_TNNT2-F	GAAGTTCGACCTGCAGGAAA
Hsa_TNNT2-R	TTCCCACGAGTTTTGGAGAC
Hsa_ACTN2-F	GAAGCACAAGCCACCCAAGG
Hsa_ACTN2-R	CACCAGCAATATCCGACACCA
Hsa_SOX2-F	GGGAAATGGGAGGGGTGCAAAAGA
Hsa_SOX2-R	TTGCGTGAGTGTGGATGGGATTGG
Hsa_OCT4-F	AGCGAACCAGTATCGAGAAC
Hsa_OCT4-R	TTACAGAACCACACTCGGAC
Hsa_VCAN-F	GTGACTATGGCTGGCACAAATTCC
Hsa_VCAN-R	GGTTGGGTCTCCAATTCTCGTATTGC

Supplementary Video 1. Spontaneous beating EHTs constructed from neonatal rat cardiac cells.

Supplementary Video 2. Calcium transients in neonatal rat cardiomyocytes under NGNF treatment.

Supplementary Video 3. Calcium transients in neonatal rat cardiomyocytes under HGHF treatment.

Supplementary Video 4. Calcium transients in neonatal rat cardiomyocytes under HGHF+VCAN treatment.

Supplementary Data 1. RNA-Seq data of rat EHTs under NGNF or HGHF treatment.

Supplementary Data 2. RNA-Seq data of NOs under NGNF or HGHF treatment.

Supplementary Data 3. RNA-Seq data of human EHTs under NGNF or HGHF treatment.

Supplementary Data 4. RNA-Seq data of human EHTs under individual culture or NO co-culture with HGHF treatment.
